# Surgical Pathology and Therapeutics, and Operative Surgery

**Published:** 1867-11

**Authors:** 


### Surgical Pathology and Therapeutics, and Operative Surgery.

*Treatment of Incised Wounds with a view to Union by the First Intention.*—The doctrine was taught by John Hunter and his pupils that the blood effused from an incised wound often constituted the bond of union. But Prof. Syme, in 1825, *(Ed. Medical Journal,)* asserted that the retention of blood between cut surfaces was the great obstacle to their adhesion. The means advised by the Professor to procure union by the first intention were "to delay the closure of the wound until the oozing from it appeared to have ceased; to apply pressure along the course of its sides, and to place some bibulous material over the lips." In a recent article *(Lancet,* July 6, 1867,) Prof. Syme states: "The observations of M. Pasteur with regard to the effect of atmospheric atoms in causing decomposition, which have led to Prof. Lister's treatment of wounds and abscesses, now established in Glasgow and Edinburgh, and certainly the most important improvement in surgical practice of recent times, has led to a complete revolution of ideas on the subject.

In cases of bruise, fracture, dislocation, and even operations of tenotomy, large quantities of blood are frequently effused more or less deeply under the integuments without causing any bad effect, and quickly disappear by means of absorption. How, then, does it happen that blood collected in the cavity of a wound should be productive of so much mischief? It can only do so, as Mr. Lister has shown, through the decomposing influence of atmospheric air, loaded with its myriads of organic atoms, and, therefore, if protected from this agency, would be no more hurtful than in the circumstances just mentioned. He has accordingly found, as stated in the preceding numbers of this journal, that wounds of the most formidable character may be divested of all their alarming features by means of carbolic acid, applied so as to prevent the impure air from entering.

This remarkable fact has led me to consider the expediency of resorting more frequently than heretofore to the use of "torsion" for the suppression of hemorrhage.

Prof. S. now thinks that *torsion* "may in many, if not all cases, be employed with advantage, instead of the ligature. In order to perform the process effectually, it is necessary that the artery should be seized by catch-forceps, and twisted until they become loose. It has been alleged that such a liberty with the vessel must cause it to slough, and thus disturb the adhesive action. But as this objection is altogether theoretical and contradicted by experience, is is unworthy of notice.

He relates two cases to substantiate the facts:

"1st. That torsion effectually restrains the hemorrhage of ordinary sized arteries.

"2d. That its action upon them does not prevent union by the first intention.

"3d. That protection from the air prevents decomposition of the blood."

